# P-422. Incidence, Risk Factors, Etiology, and Clinical Outcomes of Pneumonia in Adults with Cancer at a Tertiary Care Centre: a One Year Prospective Cohort Study

**DOI:** 10.1093/ofid/ofae631.623

**Published:** 2025-01-29

**Authors:** Simran Malik, Sanjay Bhattacharya, Sangeeta Das Bhattacharya

**Affiliations:** Indian Institute of Technology Kharagpur, Kharagpur, West Bengal, India; Tata medical center kolkata, Kolkata, West Bengal, India; Indian Institute of Technology Kharagpur, Kharagpur, West Bengal, India

## Abstract

**Background:**

Pneumonia is an important cause of morbidity, mortality, hospital admission, and healthcare-associated costs in cancer patients. We investigated the incidence, risk factors, etiology, and clinical outcomes of pneumonia in adults with cancer admitted to the ICU in Tata Medical Center Kolkata from October 2022 to September 2023.

**Figure 1:**
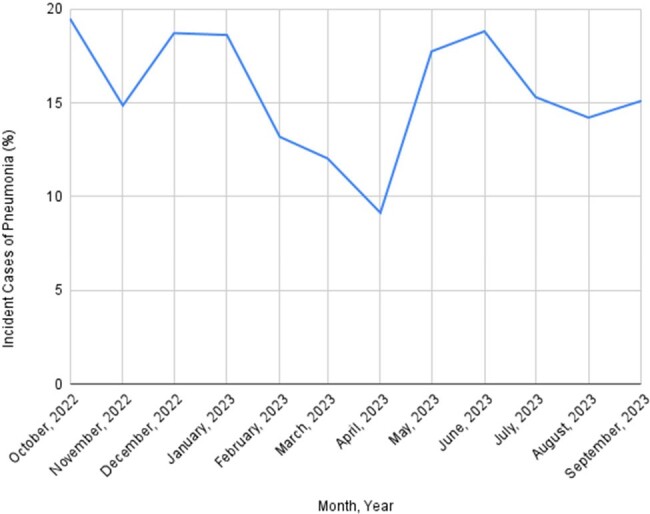
Monthly Trend in Case Identification

**Methods:**

We conducted a prospective cohort study involving two groups with malignancy admitted to the ICU: one with pneumonia and one without. Pneumonia was identified by clinical and radiological features.

Incidence was calculated as the proportion of patients in the ICU who developed pneumonia in one year. Community-acquired (CAP), healthcare-associated (HAP), and ventilator-associated pneumonia (VAP) were defined by NHSN and HAI Surveillance in India guidelines. Relative risks were calculated and logistic regression performed to assess risk factors for pneumonia and mortality; and clinical outcomes were compared using Z-tests on *STATA-17*.

**Figure 2:**
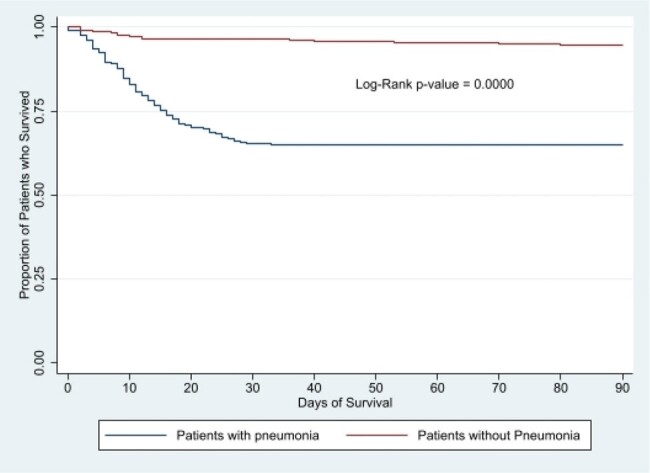
Kaplan-Meier Plot for the survival analysis for patients with or without pneumonia

**Results:**

355 individuals with pneumonia and 356 without were enrolled among 2279 in-patients. The incidence of pneumonia was 15.6% (95% CI: 14.1-17.1%). 51.8% were CAP and 48.2% were HAP. 17.9% of HAP (7.3% of all pneumonia) was VAP.

Hematological malignancies (RR=2.08), diabetes (1.31), COPD (1.78), renal dysfunction (1.73), neutropenia (1.83), smoking (1.28), chemotherapy (2.01), radiotherapy (1.56), immunotherapy (1.61), and bone marrow transplantation (2.05) increased risk for pneumonia.

258 (72.7%) cases had a positive microbiology. Of these, 123 were polymicrobial (47.7%), termed “coinfections/ superadded infections” (table 1).

All-cause 90-day mortality (39.2%), mean length of hospital stay (18.6 days), ICU stay (10.9 days), and mechanical ventilation (2 days) were higher in patients with pneumonia than those without (p< 0.05, figure 2). CAP (RR=1.42), hematological malignancies (1.59), renal dysfunction (1.51), neutropenia (1.74), chemotherapy (1.60), immunotherapy (1.55), days of ICU stay (logit OR=1.04), days of mechanical ventilation (logit OR=1.03), and co-infections/ superadded infections (logit OR=1.82) increased risk of death in pneumonia.

**Table 1:**
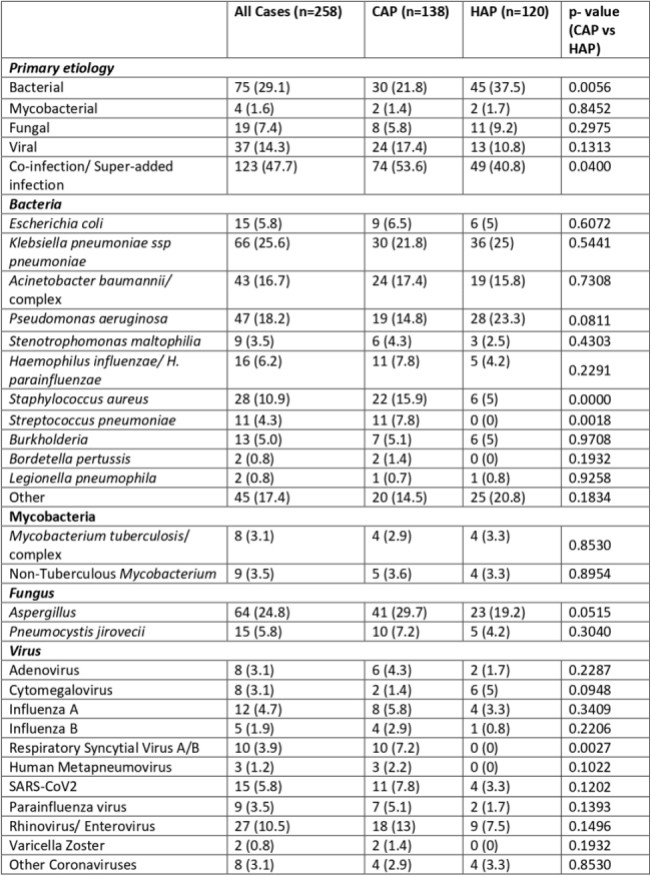
Etiology of pneumonia with positive microbiology segregated by type of pneumonia.

The denominator corresponds to the number of cases in each category that had positive microbiology.

**Conclusion:**

Pneumonia in cancer patients admitted to the ICU is associated with worse clinical outcomes.

**Table 2:**
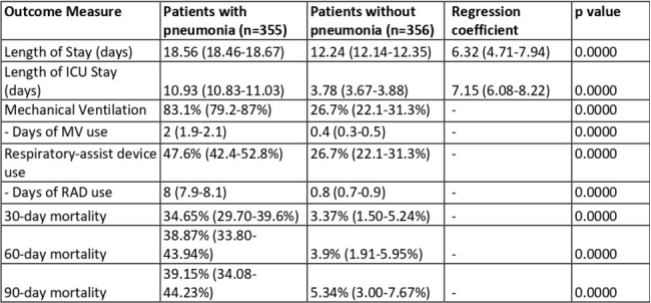
Clinical Outcome in patients with or without pneumonia

**Disclosures:**

**All Authors**: No reported disclosures

